# Edaphic and host plant factors are linked to the composition of arbuscular mycorrhizal fungal communities in the root zone of endangered *Ulmus chenmoui* Cheng in China

**DOI:** 10.1002/ece3.5446

**Published:** 2019-07-17

**Authors:** Juan Song, Long Chen, Fengmao Chen, Jianren Ye

**Affiliations:** ^1^ Collaborative Innovation Center of Sustainable Forestry in Southern China, College of Forestry Nanjing Forestry University Nanjing China; ^2^ Institute of Forest Protection, College of Forestry Nanjing Forestry University Nanjing China

**Keywords:** arbuscular mycorrhizal fungi, diversity, high‐throughput sequencing, soil properties, *Ulmus chenmoui* Cheng

## Abstract

Despite the importance of arbuscular mycorrhizal fungi (AMF) within deciduous forest ecosystems, we know little about how natural AMF communities are structured in the root zone of the endangered elm species *Ulmus chenmoui*. In this study, three *U. chenmoui* sampling sites, differing with respect to plant health, age, and growth status, were selected in Anhui Province, China. AMF biodiversity in the root zones of individual *U. chenmoui* trees was investigated using high‐throughput sequencing. In total, 61 AMF operational taxonomic units were detected. Five genera, namely *Glomus* (62.82%), *Paraglomus* (17.82%), *Rhizophagus* (4.29%), *Septoglomus* (4.06%) and *Funneliformis* (2.35%), and 29 species of AMF were identified. Correlation analysis indicated that available soil phosphorus and potassium concentrations were the main edaphic factors influencing AMF community structure. There was a difference in AMF species richness among the three *U. chenmoui* stands. Our study showed that soil nutrient concentrations and plant health, age, and growth status can exert a selective effect on the composition of the AMF population in the soil in the root zones of *U. chenmoui* trees.

## 1 INTRODUCTION

*Ulmus chenmoui* Cheng (Ulmaceae) is an endangered tree species which is endemic to eastern China and mainly found on Langya Mountain, Anhui Province, and Huashan Mountain, Jiangsu Province, in eastern China (Duan, Yong‐Fu, Yang, & Wang, 2016; Geng et al., 2016). The species is a small deciduous elm tree that is listed as “Endangered” in the China Red Data Book, based on International Union for Conservation of Nature criteria (Fu & Jin, 1992). This species can reach 15 m in height, and it is part of the outstanding scenery of the subtropical limestone mountain forests, as well as being one of the components of the natural ecosystem of Langya Mountain (Chai, Zhang, Wang, & Feng, 2007; Geng et al., 2016). Because of pressures caused by increasing intensity of tourism development in the area in recent years, the original habitat of *U. chenmoui* has been largely destroyed. At present, the natural distribution of *U. chenmoui* is restricted and there is a real risk of its extinction. Given that the seeds of this species are not easy to germinate, the seedling survival rate is low and natural renewal by understory seedlings is low, increased planting of *U. chenmoui* is of great significance in conserving this species and protecting the natural forest ecosystem.

Arbuscular mycorrhizal fungi (AMF) are the most common microsymbionts of plant roots. The AMF symbiosis is widespread in nature (Brundrett & Tedersoo, 2018; Van der Heijden, Martin, Selosse, & Sanders, 2015) and acts as an extension of the plant root system, leading to improved plant growth and reproduction through a wide variety of mechanisms, including increased nutrient availability, movement dynamics and partitioning (Marschner & Dell, 1994; Rubin, Groenigen, & Hungate, 2017), enhanced water‐use efficiency, increased abiotic stress tolerance and pathogen resistance, and greater aggregate formation in soils (Lewandowski, Dunfield, & Antunes, 2013; Marschner & Dell, 1994; Schützendübel & Polle, 2002). The nature of the interactions between different AM fungal taxa and plant hosts is not well understood, which restricts our ability to use AMF predictably (Holste, Kobe, & Gehring, 2017).

Generally, broad‐leaved deciduous forest soils have near‐neutral pH and limited available nutrients (Liu, Fox, & Xu, 2000). The presence of appropriate AMF may be essential for the survival and establishment of deciduous forest trees (Zangaro & Moreira, 2010). A diverse AMF community is important for the development and maintenance of tree growth over a range of host tree species and across varied habitats within the forest (Van der Heijden et al., 1998).

The objective of this work was to achieve a deeper understanding of the AMF species present in the root zone of *U. chenmoui* trees exhibiting different growth, health, and age statuses in the Langya Mountain, Chuzhou, Anhui Province, China. Our main questions were as follows: (a) Which AMF species are found in the root zone of the *U. chenmoui* plants? (b) Are the AMF communities homogeneous along the growth and health status gradient of the trees? and (c) How does the AMF community respond to variations in the chemical, physical, and biological properties of the soil?

## 2 MATERIALS AND METHODS

### 2.1 Study site and host plants

**Figure 1:**
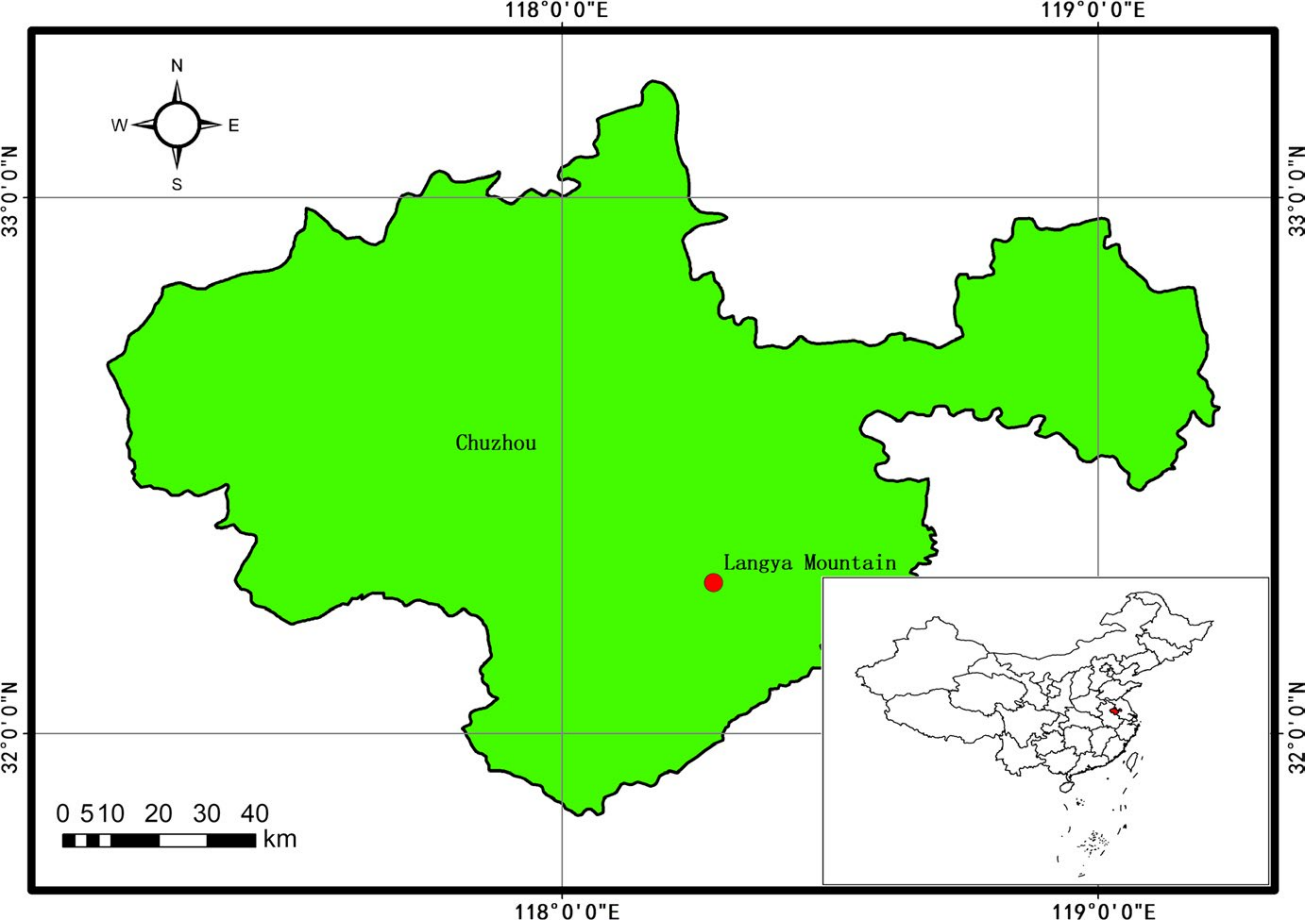
Location of the study area on Langya Mountain, Chuzhou, Anhui Province, China

The study was performed on Langya Mountain, Chuzhou, Anhui Province, China (32°16.5506′N, 118°16.5685′E; Figure 1). Samples of soil were collected in October 2015 from each of the three *U. chenmoui* sites. The trees in all three sites were natural populations, with the sites being located adjacent (within 4 km distance) to one another. The three sampling sites were LY‐1 (≥10‐year‐old elm forest, trees growing well), LY‐2 (≥10‐year‐old elm forest, trees growing poorly), and LY‐3 (≥30‐year‐old elm forest, trees growing well). Replicate trees sampled within a single site were at least 1 km apart from one other. The sampling did not follow a regular grid because we wanted to characterize the fungal communities closely associated with this dominant tree species, so samples were collected from the root zone of nine replicate individual trees at each sampling site. Each of the 27 soil samples consisted of 1 kg soil collected from the topsoil (0–15 cm depth) around an individual elm tree, using disposable equipment to avoid cross‐contamination, and subsamples (approximately 500 g) were stored under airtight conditions at either 4°C (to evaluate mycorrhizal colonization and for analysis of soil physical and chemical properties) or −80°C (for subsequent RNA or DNA isolation).

### 2.2 Mycorrhizal colonization

Only roots attached to the main roots were sampled. All collections were carefully washed in tap water and cleared by immersion in 10% (w/v) KOH for 4 hr at 90°C. The roots were then immersed in alkaline H_2_O_2_ solution (10 ml 30% (v/v) H_2_O_2_ plus 3 ml 30% NH_4_OH plus distilled water to make up to 600 ml) at room temperature for 20 min, then washed in tap water, before being immersed in 1% (v/v) HCl for 1 hr, then washed in tap water. Finally, the roots were stained in 0.05% (w/v) Trypan Blue for 4 hr at 90°C, then transferred to pure lactic acid overnight to remove excess dye, to enable visualization of the fungal structures inside the roots. Material stained in this way was cut into 1 cm long pieces and mounted on slides in pure lactic acid (~100 root pieces per tree). AM colonization of the roots was quantified under a light microscope using the intersect method (Biermann & Linderman, 1981; Phillips & Hayman, 1970). Percentage AM fungal colonization was calculated as follows: % fungal colonization = 100 × (number of intersections with a fungal structure/total number of intersections counted).

### 2.3 AM fungal spore extraction

AM fungal spores were extracted from 50 g air‐dried soil with distilled water via wet sieving (100–400 μm mesh size) and decanting methods, followed by sucrose density centrifugation (Daniels & Skipper, 1982; Ianson & Allen, 1986). The spores were collected on filter paper (Daniels & Skipper, 1982) and counted under a light microscope (ZEISS AXIO Imager M 2; Zeiss) at 40× magnification, with a sporocarp being recorded as one unit (Eugenia, Daniel, & Alejandrag, 2009).

### 2.4 Soil chemical analysis

Soil from the root zone of *U. chenmoui* trees was collected from each of the 27 plants, representing nine replicates from each of the three sites, coded LY‐1, LY‐2, and LY‐3. Each soil sample was analyzed for soil moisture, total nitrogen (N), available phosphorus (P), available potassium (K), organic matter concentration, and pH. Analyses were performed following standard protocols after soils had been air‐dried and sieved through a 2‐mm sieve. Soil pH was determined using a pH electrode in a 1:10 soil/deionized water mixture. Soil organic carbon concentration was estimated after combustion of samples at 550°C (Rossell, Gasparoni, & Galantini, 2001), available P concentration was determined using the molybdenum blue‐ascorbic acid method (Olsen, 1954), total N concentration was estimated following the Kjeldahl method (Bremner & Mulvaney, 1982), and the available K concentration was determined using the flame photometry method (Gammon, 1951). Soil moisture concentration was determined gravimetrically by oven‐drying fresh soil at 105°C overnight (Deng, Wang, Li, Zhao, & Shangguan, 2016). Soil particle density was determined using the excavation method, using expanding foam (Page‐Dumroese, Jurgensen, Brown, & Mroz, 1999).

### 2.5 Glomalin‐related soil protein (GRSP) determination

Easily extractable GRSPs (EE‐GRSPs) and total GRSPs (T‐GRSPs) were extracted from the 27 soil samples from LY‐1, LY‐2, and LY‐3, using the procedures described by Wright and Upadhyaya (1996). For EE‐GRSPs, 2 g air‐dried soil was extracted with 16 ml 20 mM citrate solution (pH 7.0) in a centrifuge tube by autoclaving at 121°C and 15 p.s.i. for 30 min. The extract was centrifuged at 6,000 × *g* for 15 min to remove soil particles. For T‐GRSPs, 2 g air‐dried soil was extracted with 16 ml 50 mM citrate solution (pH 8.0) by autoclaving at 121°C and 15 p.s.i. for 60 min. The extract was centrifuged at 6,000 × *g* for 15 min to remove soil particles. Each extraction procedure (for EE‐GRSPs or T‐GSRPs) was repeated until the supernatant was clear, and all supernatants from the one sample were combined. The protein concentration of EE‐GRSPs and T‐GRSPs in the supernatant was determined using the Bradford Coomassie Blue assay, with bovine serum albumin as the standard (Bedini et al., 2009; He, Li, & Zhao, 2010).

### 2.6 DNA extraction, PCR amplification, and high‐throughput sequencing

DNA was extracted from 500 mg freeze‐dried and milled soil samples using the Fast Soil DNA Isolation Kit (MOBIO Laboratories Inc), according to the manufacturer's instructions. The fungal 18S ribosomal RNA genes were amplified by a nested PCR reaction. GeoA2 (5′‐CCAGTAGTCATATGCTTGTCTC‐3′) and AML2 (5′‐GAACCCAAACACTTTGGTTTCC‐3′) were used as primers in the first‐round PCR, with NS31F (5′‐TTGGAGGGCAAGTCTGGTGCC‐3′) and AMDGR (5′‐CCCAACTATCCCTATTAATCAT‐3′) primers being used in second‐round PCR, where the reaction used specific primers with multiplex‐identifying barcodes and partial adapters for sequencing. All PCR amplifications were performed in triplicate, in a 50 μl reaction mixture containing 2 μl (30 ng) template DNA, 4 μl dNTPs (2.5 mM each), 2 μl of each primer (10 μM), 5 μl 10× Pyrobest buffer (TaKaRa), 0.3 μl Pyrobest DNA polymerase (DR005A; 2.5 U/μl, TaKaRa), and 34.7 μl ddH_2_O. The first‐round PCR was carried out using the following procedure: 94°C for 2 min, followed by 30 cycles at 94°C for 30 s each, 59°C for 1 min, and 72°C for 2 min and a final extension at 72°C for 10 min. The procedure for the second‐round PCR reaction was 94°C for 5 min, followed by 35 cycles at 94°C for 30 s each, 58°C for 45 s, and 72°C for 45 s and a final extension at 72°C for 10 min. The second‐round reaction products were run on a 1% (w/v) agarose gel, voltage 170 V for 30 min, and the size of the amplicon was confirmed to be ≥250 bp.

The amplicons were extracted from 2% agarose gels and purified using the AxyPrep DNA Gel Extraction Kit (Axygen Biosciences), according to the manufacturer's instructions, and quantified using QuantiFluor™‐ST (Promega). Purified amplicons were pooled in equimolar concentrations and were paired‐end sequenced (2 × 300) on an Illumina MiSeq platform (Majorbio) according to standard protocols (Caporaso et al., 2010). The raw reads were deposited into the NCBI Sequence Read Archive (SRA) database (Accession Number: SRP149304).

### 2.7 Sequencing data analysis

The raw fastq files (bioinformatics.babraham.ac.uk/projects/fastqc) were demultiplexed, quality‐filtered by Trimmomatic (Bolger, Lohse, & Usadel, 2014), and merged by FLASH to Improve Genome Assemblies (version 1.2.7; Magoc & Salzberg, 2011) with the following criteria: (a) the 300‐bp reads were truncated at any site receiving an average quality score <20 over a 50‐bp sliding window, discarding the truncated reads that were shorter than 50 bp; (b) the barcodes and primers had to match exactly, with reads containing ambiguous characters being discarded; (c) only sequences that had an overlap length of more than 10 bp were assembled; (d) chimera checking was conducted by USEARCH (Edgar, 2010) software. Reads which could not be assembled were discarded. After quality filtering and chimera removal, high‐quality clean tags were obtained. Finally, operational taxonomic units (OTUs) were clustered with 97% similarity cutoff using UCLUST (Edgar, 2010). The representative sequences were then blasted against the NCBI nucleotide database using Blast2GO (Conesa et al., 2005) to identify and remove non‐AMF sequences. Taxonomic classification was assigned to fungal OTUs from the SILVA database (Quast et al., 2013).

The sequences were clustered into OTUs, and the AM fungal community structure of each sample was determined on the basis of the sequences obtained within the subphylum Glomeromycotina. To calculate the richness and diversity indices (Cole et al., 2009; Schloss, Gevers, & Westcott, 2011), Shannon, Chao1, Observed_species, Good's_coverage and PD_whole_tree (qiime version v.1.8 http://qiime.org/scripts/alpha_rarefaction.html) were calculated.

### 2.8 Statistical analysis

Statistical analyses were carried out to compare variables at the three sites (LY‐1, LY‐2, and LY‐3). Analysis of mycorrhizal colonization, EE‐GRSP and T‐GRSP concentrations, spore densities of AM fungal communities, and soil physical and chemical characteristics were carried out using parametric one‐way analysis of variance (ANOVA). Where ANOVA was significant and the variances were equal, multiple comparisons among the mean values of the different sampling sites were carried out using the Tukey Honestly Significance Difference test; if the variances were unequal, Tamhane's T2 post hoc tests were used to determine the significance of any differences between sampling sites. The analyses were carried out using SPSS Statistics v.19 software (IBM).

The relative contribution of the soil factors to AMF community composition was determined using ordination analysis. Detrended correspondence analysis (DCA) was first carried out to decide between linear or unimodal response models for AMF species data, with regard to our short‐length gradients in DCA analysis (length of gradient < 3), the linear model of canonical redundancy analyses (RDA) then being selected as the appropriate model for the data set. All data were log (*x* + 1) transformed in order to reduce the influence of common taxa. Furthermore, data were centered and standardized by species to harmonize different scales (Leps & Smilauer, 2003). The type II scaling method was used, and the cosine of the angle between the vectors denoted their (linear) correlation. The statistical significance of the regression was evaluated using the Monte Carlo‐based permutation test (MCP), based on a significance level of 0.05 (Abdelfattah, Wisniewski, Droby, & Schena, 2016). The Monte Carlo permutation test with 499 unrestricted permutations was used to determine to what extent the soil parameters (soil available P, available K, total N concentrations, pH, moisture, soil particle density, and soil organic C concentration) could explain the soil AMF community structure. These analyses were performed using the CANOCO 4.5 software (Ter Braak & Smilauer, 2002).

## 3 RESULTS

### 3.1 Microscopic root colonization measurements, soil properties and spore densities

**Figure 2:**
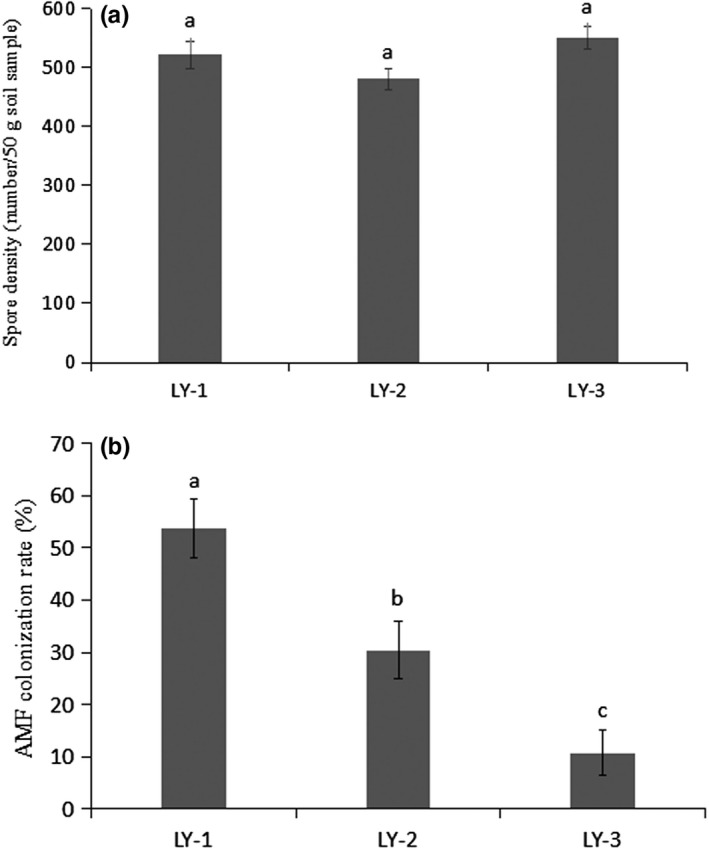
AMF spore density (a) and total colonization (b). Means with a common lowercase letter in the same column are not significantly different, *p* (perm), *p* value by permutation (*p* < 0.05)

**Figure 3:**
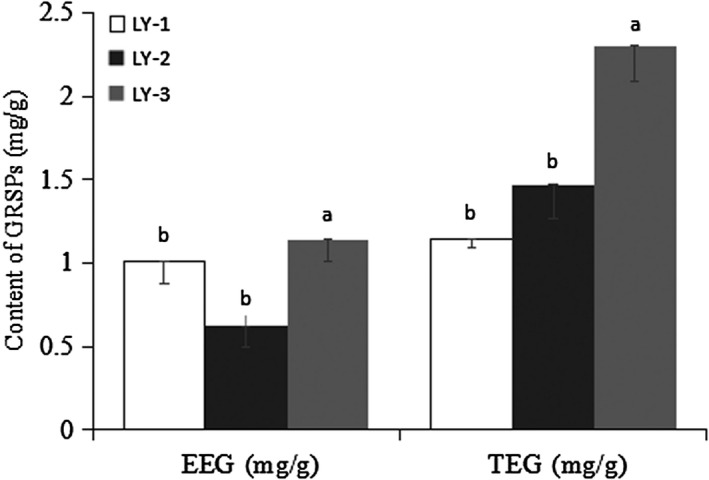
Total glomalin‐related soil protein (T‐GRSP) and easily extractable glomalin‐related soil protein (EE‐GRSP) concentrations at different study sites. Each value is the mean ± *SD* (*n* = 3). Different letters on bars for the same glomalin protein indicate statistically significant differences (*p* < 0.05) among sites

Soil pH was significantly different between the three sites (pH 6.40–8.12), with the soil from the root zone of the young healthy trees (LY‐1) having a pH significantly lower than that from the other two sites (Table 1), but soil moisture was not significantly different between the different sites. Total soil N concentration was significantly lower in LY‐1 than in the other sites. Site LY‐2, containing the young but poorly growing trees, had the lowest values of soil organic C, available P, and available K concentrations, whereas the highest concentrations for both available P and K were found in LY‐1 (Table 1). Soil particle density and spore density values were not significantly different between the three sites (Figure 2a). With respect to EE‐GRSP concentration, the value in LY‐3, the site with older trees growing well, was significantly higher than that in LY‐2, whereas, for T‐EGRSP concentration, the value in LY‐3 was significantly higher than that in both LY‐1 and LY‐2 (Figure 3). The mean ± *SD* AM fungal colonization rate ranged from (10.75 ± 4.27%; LY‐3) to (30.38 ± 5.50%; LY‐2) and (53.75 ± 5.51%; LY‐1), with the values for each of the sites being significantly different from the others (Figure 2b).

**Table 1:** Soil chemical properties, spore densities, and mycorrhizal colonization rate (mean ± *SD*) for each site

Site category	Organic C (%)	Total N (%)	P (mg/kg)	K (mg/kg)	Moisture content (%)	Soil particle density (kg/L)	Soil pH
LY‐1	3.13 ± 0.46ab	0.15 ± 0.02b	62.80 ± 2.99a	117.89 ± 0.57a	11.09 ± 0.47a	1.08 ± 0.27a	6.40 ± 0.37b
LY‐2	2.69 ± 0.51b	0.52 ± 0.03a	32.10 ± 1.80b	61.69 ± 0.71b	13.09 ± 1.01a	1.11 ± 0.53a	8.12 ± 0.19a
LY‐3	4.15 ± 0.15a	0.50 ± 0.03a	56.41 ± 3.85a	56.86 ± 1.84c	12.48 ± 0.69a	1.07 ± 0.40a	7.60 ± 0.40a

Note

Means with a common lowercase letter in the same column are not significantly different, *p* (perm), *p* value by permutation (*p* < 0.05). LY‐1: ≥10 elm forest, trees growing well, LY‐2: ≥10 elm forest, growing poorly and LY‐3: ≥30 elm forest, trees growing well.

Abbreviations: EEG, easily extractable glomalin‐related soil proteins; K, available potassium concentration; Organic C, soil organic carbon; P, available phosphorus concentration; TEG, total glomalin‐related soil proteins.

After filtering out chimeras and non‐AM fungal sequences, a total of 126,823 AM fungal reads were observed (Table 2), and 61 AMF OTUs in total were detected from the three sites. We found that site LY‐1 had the highest mean ± *SD* Shannon diversity index (*H*; 3.89 ± 0.03), while site LY‐2 had the lowest *H* value (3.16 ± 0.08). A comparison of the Chao1 values revealed the highest value in LY‐1 (60.68 ± 0.35), and the lowest in LY‐2 (55.63 ± 0.39), while the number of observed_species was found to be similar to the number of estimated OTUs (Chao1 indices; Table 2).

**Table 2:** The biodiversity index of arbuscular mycorrhizal fungi (AMF) in the three sampling sites

Sample ID	Chao1	Goods_coverage	PD_whole_tree	Shannon	Observed_species	Final_tags
LY1	60.68 ± 0.35a	0.99 ± 0.00a	2.89 ± 0.06a	3.89 ± 0.03a	58.17 ± 0.46a	42410
LY2	55.63 ± 0.39c	0.99 ± 0.00a	2.26 ± 0.05c	3.16 ± 0.08c	53.23 ± 0.23b	41989
LY3	59.10 ± 0.12b	0.99 ± 0.00a	2.66 ± 0.04b	3.63 ± 0.04b	57.28 ± 0.80a	42424

Note

LY‐1, 10‐year‐old elm forest, trees growing well; LY‐2, 10‐year‐old elm forest, trees growing poorly; LY‐3, 30‐year‐old elm forest, trees growing well. Any two samples with a common letter within a column are not significantly different (*p* > 0.05).

**Figure 4:**
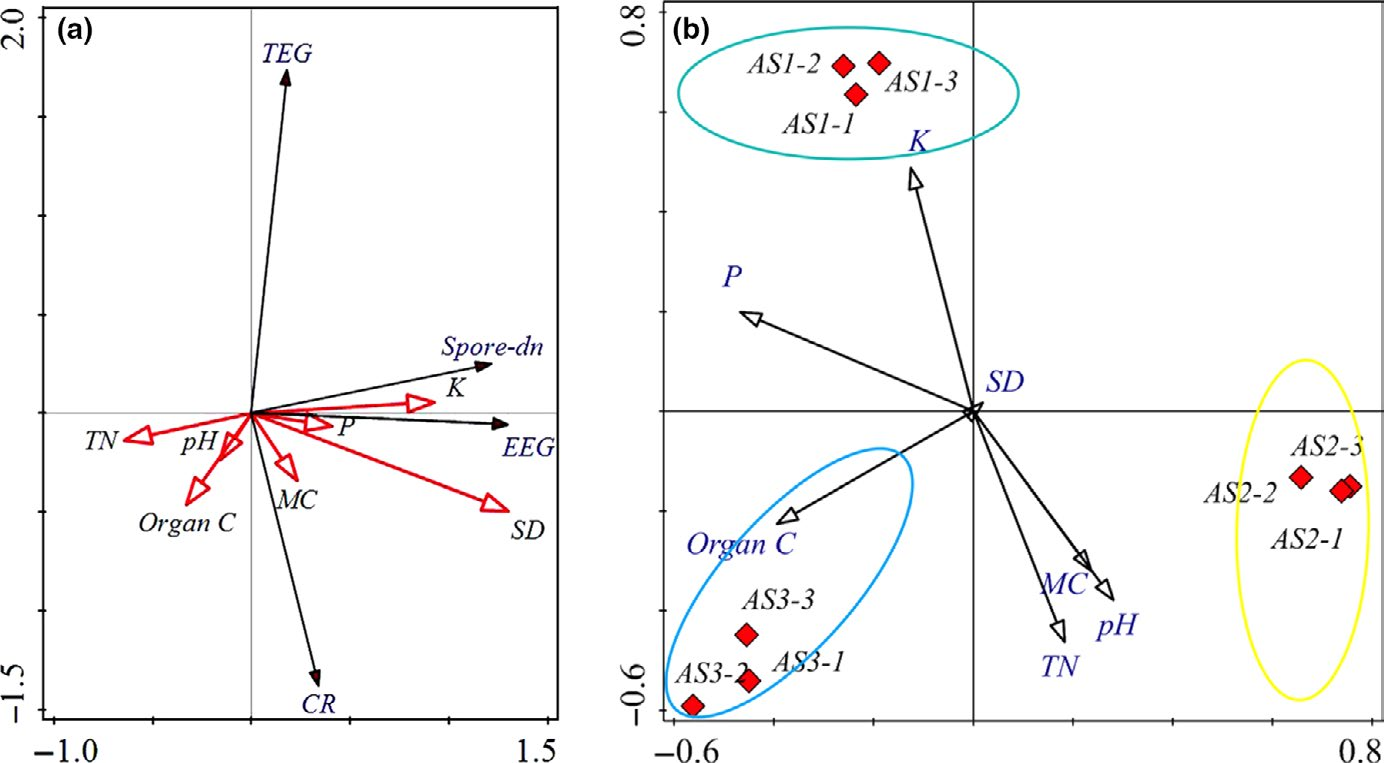
Redundancy analysis (RDA) of biological variables (CR, EEG, Spore‐dn and TEG) (a) and arbuscular mycorrhizal fungi (AMF) communities (b) changes with soil geochemical factors in the root zone of *U. chenmoui*. Abbreviations: Organic C (soil organic carbon), P (available phosphorus concentration), K (available potassium concentration), *SD* (Soil particle density), MC (Moisture content), TN (Total N), EEG (easily extractable glomalin‐related soil proteins), TEG (total glomalin‐related soil proteins), CR (Colonization rate (%)). AS1 (refers to LY‐1 AMF community structure), AS2 (refers to LY‐2 AMF community structure), AS3 (refers to LY‐3 AMF community structure). LY‐1, LY‐2, and LY‐3 represent the three sites

RDA showed the associations between the tested environmental factors and biotic variables in the root zones of *U. chenmoui* trees to be affected by differential plant health, age, and growth status. As illustrated in Figure 4a, the first two RDA axes showed high eigenvalues (0.41 and 0.16, respectively) in comparison to subsequent axes, which indicated that environmental factors had significant effects on biotic variables in the root zone of *U. chenmoui*. On the whole, the axes explained 58.2% of the variance of the biological variables–environment relationships (Figure 4a). Furthermore, RDA analysis showed significant positive correlations between spore density and available P concentration, as well as between available K concentration and soil particle density (*r* = 0.866, *r* = 0.707, *p* < 0.05). Significant positive correlations were also observed between colonization rate (%) and the variables soil organic carbon concentration, as well as between soil pH and moisture content (*r* = 0.643, *r* = 0.500, *p* < 0.05). There was also a significant negative correlation between spore density and total N concentration (*r* = −0.994, *p* < 0.05; Figure 4a).

RDA and the Monte Carlo test were performed to discern the association between the composition of the AMF community and the environmental factors (Figure 4b, Table 3). The ordination diagram indicated that available P concentration (*F* = 3.2, *p* = 0.002) contributed significantly to the variation in AMF communities, followed by the available K concentration (*F* = 2.4, *p* = 0.026). However, soil particle density (*F* = 1.7, *p* = 0.098), total N concentration (*F* = 1.4, *p* = 0.184), soil pH (*F* = 0.7, *p* = 0.678), and soil organic C concentration (*F* = 0.3, *p* = 0.772) did not contribute significantly to the variation in composition of the AMF communities (Table 3).

**Table 3:** Result of Monte Carlo permutation test for the influence of the soil K, P, SD, TN, pH, and Organ C contents on soil AMF community structure

Name	Variation explains %	Contribution %	Pseudo‐*F*	*p*
K	25.4	31.2	2.4	0.026
P	26.0	32.0	3.2	0.002
SD	12.2	15.0	1.7	0.098
TN	9.5	11.8	1.4	0.184
pH	5.0	6.2	0.7	0.678
Organ C	3.1	3.8	0.3	0.772
Soil geochemical factors (K, P, SD, TN, pH, Organ C)	81.2			

Note

K, P, SD, TN, pH, and Organ C contents indicate soil available potassium, available phosphorus, soil particle density, total N, and soil organic carbon contents, respectively.

### 3.2 AMF community composition and structure analysis

**Figure 5:**
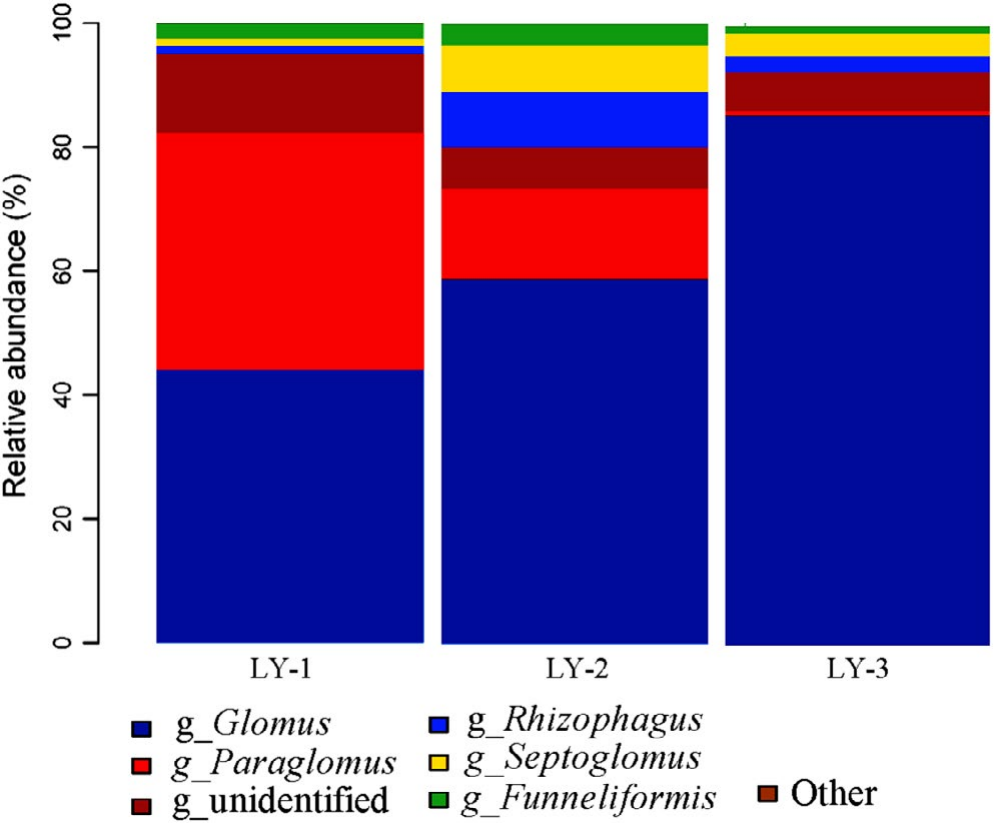
Proportion of total read numbers of operational taxonomic units (OTUs) grouped by arbuscular mycorrhizal fungal (AMF) genus in the root zone of *U. chenmoui*. LY‐1, LY‐2, and LY‐3 represent the three sites

The composition of the AMF communities at the three sampling sites and the relative abundance of OTUs were classified to the genus level. To further investigate the relative abundance values in sites LY‐1, LY‐2 and LY‐3, the most frequent genera were clustered. The dominant AMF genera across the sampling sites were *Glomus*, *Paraglomus, Rhizophagus, Septoglomus,* and *Funneliformis* (Figure 5). *Glomus* was the most frequent genus in sites LY‐2 (58.84%) and LY‐3 (85.57%), while LY‐2 was the site with the highest frequency of *Septoglomus* (7.53%). *Paraglomus* occurred in all sites, but at a much higher frequency in LY‐1 (38.16%) than in LY‐2 (14.59%) or LY‐3 (0.72%). *Rhizophagus* (8.97%) and *Funneliformis* (3.35%) performed better in LY‐2 than in LY‐1 or LY‐3 (Figure 5). The remaining components belonged to OTUs which were labeled as either “other” or “unidentified” in Figure 5. Most of the AMFs detected belonged to the Glomeraceae, with five genera and 29 species of AMF in the three sampling sites. The genus *Glomus* represented 62.82% of the total number of subphylum Glomeromycotina members, with *Glomus* being the dominant fungal genus across these samples, whereas the contributions of the other genera were lower. These other genera included *Septoglomus* (4.06%, two species), *Paraglomus* (17.82%, two species), *Rhizophagus* (4.29%, three species), and *Funneliformis* (2.35%, two species; Table 4; Figure 5). These results indicate that the method had captured a broad range of AMF species at a high resolution.

**Table 4:** AMF OTU taxa detected at the three sites

ID	LY‐1	LY‐2	LY‐3	Taxonomy
OTU_1	0.38	0.01	0.00	*s*__*Paraglomus_majewskii*
OTU_19	0.00	0.01	0.01	*s__Rhizophagus_iranicus*
OTU_59	0.00	0.01	0.00	*s__Funneliformis_caledonium*
OTU_6	0.09	0.01	0.04	s__*Glomeromycotina_sp*._AB‐2014
OTU_23	0.00	0.00	0.01	s__*Glomus_indicum*
OTU_13	0.01	0.03	0.01	s__*Glomeromycotina_sp*._MIB_8859
OTU_10	0.00	0.07	0.00	*s__Septoglomus_constrictum*
OTU_17	0.01	0.01	0.01	*s__Rhizophagus_intraradices*
OTU_16	0.01	0.01	0.01	*s__Glomeromycotina_sp*._AB‐2014
OTU_81	0.01	0.00	0.00	*s__Glomus_sp._*8_FW‐2016
OTU_11	0.01	0.01	0.09	*s__Glomus_indicum*
OTU_3	0.01	0.27	0.01	s_*Septoglomus_viscosum*

Note

This table shows only the relatively dominant OTUs. LY‐1:10‐year‐old forests (growing well); LY‐2:10‐year‐old forests (growing poorly), and LY‐3:30‐year‐old forest (growing well).

Abbreviation: s, species.

## 4 DISCUSSION

### 4.1 AMF species diversity in individual *U. chenmoui* root zones

To our knowledge, this is the first comprehensive assessment of AMF communities in *U. chenmoui*, an endangered tree endemic to eastern China. The majority of similar studies have targeted temperate annual plant species, or species of grasslands or agro‐ecosystems (Ohsowski, Zaitsoff, Opik, & Hart, 2014). Our data contribute toward a more complete understanding of the diversity and ecology of *U. chenmoui*‐associated AMF species. The present study shows that *U. chenmoui* can sustain highly diverse AMF communities.

Host plant characteristics shape AMF communities strongly (Haichar, Heulin, Guyonnet, & Achouak, 2016). Of the three sites, site LY‐1 (≥10‐year‐old elm forest with trees growing well) exhibited the greatest diversity of the AMF community in our study (Table 2). This site also exhibited the highest AMF richness and Shannon index value. It is possible that an increase in AMF OTUs could reflect an increase in those AMFs that show host preferences (Maherali & Klironomos, 2007). Our findings were consistent with those of previous reports, which showed that AMF communities in root zone soil are not random assemblages, with host plant characteristics playing a major role in the modulation of AMF community composition (Guyonnet et al., 2017; Hannula, Boer, & Veen, 2010; Hart et al., 2009; Singh, Munro, Potts, & Millard, 2007). Host plant effects on AMF community composition have been reported from several habitats, including temperate grasslands (Vályi, Rillig, & Hempel, 2015), alpine sites (Becklin, Hertweck, & Jumpponen, 2012), and boreal forests (Davison, Öpik, Daniell, Moora, & Zobel, 2011).

High‐throughput sequencing revealed distinct differences in AMF community structure (Table 4) and diversity (Figure 5) between the root zones of *U. chenmoui* from different sites. Only four or five AMF genera were associated with the root system of each *U. chenmoui* tree, based on the observation that *Glomus*, *Paraglomus*, *Rhizophagus*, *Septoglomus,* and *Funneliformis* were found to co‐exist in 91.34% of all of the 27 root zones sampled. *Glomus* was the most frequent genus in sites, with an overall frequency of 62.82% across all 27 samples. *Glomus* are thought to be better adapted to a particular environment due to their high sporulation rates (Daniell, Husband, Fiter, & Young, 2001) and their ability to rapidly colonize colonize soil (Biermann & Linderman, 1983). The prevalence of *Glomus* in all sample sites suggests that alpine habitats favor the presence of this AMF genus.

In this study, the AMF phylotypes found in the LY‐2 sample site, especially with respect to the most abundant genus, *Septoglomus*, must have some traits that allow them to adapt to the specific conditions at this site or to survive as highly adapted spores in the soil. Some studies have demonstrated that *Septoglomus* was found exclusively under extreme conditions of drought and high temperatures (Bonfim et al., 2016; Symanczik, Chwat, Boller, Wiemken, & Alyahya'Ei, 2014) and can play an important role in mitigating drought impact on plants (Grümberg, Urcelay, Shroeder, Vargas‐Gil, & Luna, 2015). Moreover, the difference in the diversity and community composition of AM fungi under the unhealthy trees in LY‐2, particularly the relatively high frequency of *Septoglomus* (7.53%), compared to sites LY‐1 (0.99%) and LY‐3 (3.67%), which both contained healthy trees, suggested that the host plants in LY‐2 may allocate different (qualitatively and/or quantitatively) carbon resources to their AM fungal partners (Aguilar‐Trigueros, Powell, Anderson, Antonovics, & Rilling, 2014), such as specific root‐exudate patterns.

The identification of individual AMF species was restricted to certain sporulating species; therefore, minor or nonsporulating species might have been neglected (Tonin, Vandenkoornhuyse, Joner, Straczek, & Leyval, 2001). Our results also revealed that *Rhizophagus* (8.97%) and *Funneliformis* (3.35%) were common genera in LY‐2; this may be because the conditions at LY‐2 (≥10‐year‐old elm forest, trees growing poorly) sites could have stimulated colonization by *Funneliformis* and *Rhizophagus* as a result of stress. This observation suggests that some AMF species are specialists, occurring only in certain ecosystems and only on the plants adapted to these ecosystems (Shukla et al., 2009; Sturmer & Siqueira, 2011).

It should be noted that many of the representative sequences did not match the sequences deposited in the SILVA 128_18S database (Table 4, Figure 5). The Earth Microbiome Project reported that the percentages of sequences obtained did not match the sequences reported to be representative of specific environments (Gilbert, Jansson, & Knight, 2014). In other words, a great number of AM fungi detected in the present study could not be identified, so were not deposited in the databases. In addition, there were a number of the AM fungi which could not be identified to the genus level due to limitations of the SILVA database, so they were classified only at the class, order, or family levels (shown as “Unclassified” or “other” in Figure 5).

Our research data suggested that the different characteristics (e.g., age, growth, and health) exhibited by plants of the same species growing under different conditions, appeared to affect the composition of the AMF community, reinforcing the importance of the symbiosis (Bever, Richardson, Lawrence, Holmes, & Watson, 2009), especially during early tree growth. The mycorrhizal colonization of roots also varied among the sites and according to the age of the trees in this study. Colonization was high in the LY‐1 trees (10‐year‐old trees, growing well) and low in the LY‐3 trees (30‐year‐old trees, growing well). This finding might be due to the fact that younger plants require the uptake of more nutrients through their root system for survival than do older plants, by which point the plant is totally independent of their fungal symbionts. DukeJackson and Caldwell (1994) and Basumatary, Parkash, Tamuli, Saikia, and Teron (2015) also reported that mycorrhizal root colonization may be affected by plantation age.

### 4.2 Effects of soil chemistry on AM fungal density and spore density

We found that the combination of biotic factors (namely plant age, health, and growth status) did not significantly affect AM fungal spore density (Figure 2a). There are many factors that could affect spore proliferation in a given host root zone, and values for AMF spore density at different sites are known to vary markedly (Koske, 1987). Soil physical and chemical properties, host dependence, age of the host plant, and distribution patterns of AMF spores in the soils have previously been reported to be among the factors involved (Bever, Morton, Antonovics, & Schultz, 1996; Engelmoer, Behm, & Toby, 2014; Qin et al., 2015). In the current study, a positive correlation between AMF spore density and available P was observed (Figure 4a), similar to that reported by Ong et al. (2012). The positive correlation may be due to the fact that the low P concentration in the soil resulted in an increase in mycorrhizal sporulation. This finding is in agreement with Muleta, Assefa, Nemomissa, and Granhall (2008), and to some other studies (Isobe, Aizawa, Iguchi, & Ishii, 2007; Mohammad, Hamad, & Malkawi, 2003), which also reported relationships between AMF spore density and some chemical properties of the soil (pH, available P, soil, and organic matter). In summary, the relationships between AMF spore density and soil chemical properties were not consistent in the present study but differed according to variation in root characteristics and community composition of members of the subphylum Glomeromycotina.

Because AMF promotes P acquisition (Smith & Read, 2008; van der Heijden, Dombrowski, & Schlaeppi, 2017), it is likely that a shift in a plant's primary source of soil P to stable organic P and inorganic P would involve a change in the primary function of specific AMF associations. Although limited, there is evidence that AMF species differ in their ability to acquire (Cavagnaro, Smith, Smith, & Jakobsen, 2005) and transport P to their host plant (Munkvold, Kjøller, Vestberg, Rosendahl, & Jakobsen, 2004), and that AMF taxa may benefit plants to different degrees, based on the type of plant and the amount of available soil P (Song et al., 2017; Yu, Xue, He, Liu, & Steinberger, 2017). Consequently, it is possible that the AMF taxa with greater relative abundance in the three sites were specialists on host plants of good health status, while those taxa with lower relative abundance were specialists on trees which were growing poorly (Table 2; Figure 5). Nonetheless, it is striking that the dominant plant taxon and the relative abundances of most AMF taxa on the mountain remained unchanged. This is interesting in the light of studies on AMF, which report wide differences in the ability of different fungal taxa to mobilize and acquire P from different sites (Rodríguez‐Echeverría et al., 2017).

## 5 CONCLUSIONS

Here, we report on the diversity and identity of arbuscular mycorrhizal fungi in the root zone of *U. chenmoui*, an endangered tree endemic to eastern China. Our results suggest that the plantation age, tree health, and available soil nutrient status were important factors with respect to modulating the composition of AMF communities. AM fungal colonization rate was observed to decrease with tree age. As a result, the relationships between AMF community composition and soil properties may become particularly relevant in the future. More importantly, the results of this study indicated that *U. chenmoui* exhibits marked AMF biodiversity. The identification and characterization of the as‐yet unnamed AMF species associated with specific *U. chenmoui* sites could facilitate the design of specific AMF inocula and application schemes for mycorrhization work with *U. chenmoui* saplings, with the purpose of developing improved sustainable forestry practices. Future studies will have to be conducted to show whether, over longer periods of time, these patterns become even more pronounced, with increased AMF species diversity and a clear shift toward forestry‐adapted species of the Glomeromycotina.

## CONFLICT OF INTEREST

The authors declare that they have no conflicts of interest in this work. We declare that we do not have any commercial or associative interest that represents a conflict of interest in connection with the work submitted.

## AUTHOR CONTRIBUTIONS

Fengmao Chen and Juan Song conceived and designed the experiments. Fengmao Chen, Juan Song, and Long Chen performed the experiments. Juan Song and Long Chen analyzed the data and drafted the manuscript. Fengmao Chen, Juan Song, Long Chen and Jianren Ye participated in experimental coordination and revision of the manuscript. Fengmao Chen and Juan Song proofread and finalized the manuscript, and all authors agreed to the submission of the final draft of the manuscript. We sincerely thank Professor Hui Sun, Qingfang He and Associate Professor Ake Liu for editorial comments and data analysis.

## Data Availability

We declare that we have deposited our data on the National Coalition Building Institute (NCBI) database. The raw reads were deposited into the NCBI Sequence Read Archive (SRA) database (Accession Number: SRP149304), BioProject ID: PRJNA473467; BioSample accessions SAMN09273686, SAMN09273687, SAMN09273688.
